# Molecular Characterization of U-box E3 Ubiquitin Ligases (TaPUB2 and TaPUB3) Involved in the Positive Regulation of Drought Stress Response in *Arabidopsis*

**DOI:** 10.3390/ijms222413658

**Published:** 2021-12-20

**Authors:** Jae Ho Kim, Moon Seok Kim, Dae Yeon Kim, Joseph Noble Amoah, Yong Weon Seo

**Affiliations:** Department of Plant Biotechnology, Korea University, Seoul 02841, Korea; jhkim169@korea.ac.kr (J.H.K.); kms7421322@korea.ac.kr (M.S.K.); dykim@korea.ac.kr (D.Y.K.); jamoah@korea.ac.kr (J.N.A.)

**Keywords:** wheat, plant U-box E3 ligase, drought, ubiquitination, TaPUB2, TaPUB3

## Abstract

Plant U-box E3 ubiquitin ligase (PUB) is involved in various environmental stress conditions. However, the molecular mechanism of U-box proteins in response to abiotic stress in wheat remains unknown. In this study, two U-box E3 ligase genes (*TaPUB2* and *TaPUB3*), which are highly expressed in response to adverse abiotic stresses, were isolated from common wheat, and their cellular functions were characterized under drought stress. Transient expression assay revealed that TaPUB2 was localized in the cytoplasm and Golgi apparatus, whereas TaPUB3 was expressed only in the Golgi apparatus in wheat protoplasts. Additionally, TaPUB2 and TaPUB3 underwent self-ubiquitination. Moreover, TaPUB2/TaPUB3 heterodimer was identified in yeast and the cytoplasm of wheat protoplasts using a pull-down assay and bimolecular fluorescence complementation analysis. Heterogeneous overexpression of *TaPUB2* and *TaPUB3* conferred tolerance to drought stress. Taken together, these results implied that the heterodimeric form of U-box E3 ubiquitin ligases (TaPUB2/TaPUB3) responded to abiotic stress and roles as a positive regulator of drought stress tolerance.

## 1. Introduction

Plants are consistently exposed to adverse environmental conditions throughout their life cycle. They are regularly subjected to harmful environmental cues that affect their growth, development, and yield. Therefore, plants have evolved various physiological, biochemical, and molecular strategies to cope with these extreme environmental conditions [1,2]. Although the metabolic response to drought stress in various crops has been widely studied, little is known about the molecular mechanisms underlying drought tolerance, especially in wheat. Therefore, the study of stress responsive genes in wheat would clarify its response to various conditions.

Wheat (*Triticum aestivum* L.) is an economically important cereal crop in the developing world, as an estimated 80 million farmers depend on wheat for their livelihood [3]. Recurrent droughts caused by climate change are a major constraint on the global wheat productivity [4]. Recently, numerous efforts have been made to mitigate drought stress by breeding drought-resistant varieties. However, wheat has a large and structurally complex genome, and drought tolerance in wheat is a complex trait that is controlled by many genes. To date, several candidate genes for drought stress tolerance in wheat have been reported. *TaMYB31-B* overexpression in *Arabidopsis* has been shown to regulate the drought stress response in an abscisic acid (ABA)-dependent manner [5]. TaAREB3 (ABA response element binding 3), a new member of the AREB transcription factor family, not only enhances drought tolerance, but also strengthens ABA sensitivity and cold tolerance [6]. Moreover, wheat E3 ligase (*TaSAP5*) is highly induced by drought stress and ubiquitinates defective ribosomal products, which are negative regulators of DREAB2A, by promoting their degradation via the 26S proteasome pathway [7]. Furthermore, the overexpression of *TaFBA1* was reported to be a positive response to drought stress, and it increases reactive oxygen species (ROS) scavenging, which improves plant tolerance to drought conditions [8].

Post-translational modifications play a crucial role in plant metabolism by modulating transport, cell fate and development, structural support, and transcription [9]. The ubiquitin (Ub)-proteasome system (UPS) plays a vital role in the regulatory mechanism of protein ubiquitination and degradation in plants. The UPS degrades a wide range of proteins in plant organelles, thereby regulating growth, hormone signaling, and abiotic stress responses [10]. In this process, mono- or poly-ubiquitin attaches to the substrate by E1 (ubiquitin-activating enzyme), E2 (conjugating enzyme), and ubiquitin E3 ligase. Among these, E3 ligase regulates an essential rule of the induced target substrate gene or protein level.

In plants, E3 ligases can be divided into four main classes depending on the presence of HECT (HOMOLOGY TO E6-AP *C*-TERMINUS), RING (REALLY INTERESTING NEW GENE)/PUB (Plant U-box), SCF (SKP1-CULLIN-F-box), and APC (Anaphase-Promoting Complex) [11]. The U-box protein was first isolated in yeast; it possesses E3 ligase activity and comprises approximately 70 amino acids [12,13]. PUB genes have previously been identified, and mutations (such as deletions) in the U-box domain have been found to reduce E3 ligase activity [14,15]. PUBs have been discovered in the genomes of many plant species. For example, a recent genome-wide analysis revealed 66, 77, and 213 PUBs in *Arabidopsis thaliana* (*AtPUB*) [16], rice (*OsPUB*) [17], and wheat (*TaPUB*) genome, respectively [18]. U-box-ARM (armadillo)-encoded proteins have been implicated in the regulation of various biotic and abiotic pathways, such as cell death, defense response, cold stress, and salt stress pathways [19,20,21]. U-box proteins also affect other pathways; they play important roles in self-incompatibility [22], pseudo-self-incompatibility [23], hormone regulation [24], and plant development [25]. U-box-containing genes have previously been identified in wheat [26], but little is known about their physiological role in drought tolerance. Therefore, understanding signal transduction in wheat during drought conditions will be helpful in increasing the pace of molecular breeding of drought-tolerant wheat cultivars.

In this study, we identified two U-box-type ubiquitin E3 ligases in wheat (*Triticum aestivum* L. ‘Keumkang’) TaPUB2 and TaPUB3. *TaPUB2* and *TaPUB3* were characterized in response to abiotic stress. Moreover, the yeast two-hybrid assay and bimolecular fluorescence complementation (BiFC) assay elucidated the heterodimeric complex formed by TaPUB2 and TaPUB3. Finally, we overexpressed *TaPUB2* and *TaPUB3* in *Arabidopsis* and showed their phenotypic effects under drought stress condition. Although further studies are needed to elucidate the function of the two heterodimer-forming genes (*TaPUB2* and *TaPUB3*)*,* our study might contribute to future work on understanding the molecular mechanism of TaPUB2/TaPUB3.

## 2. Results

### 2.1. Isolation of Drought-Induced PUB Genes

**Figure 1:**
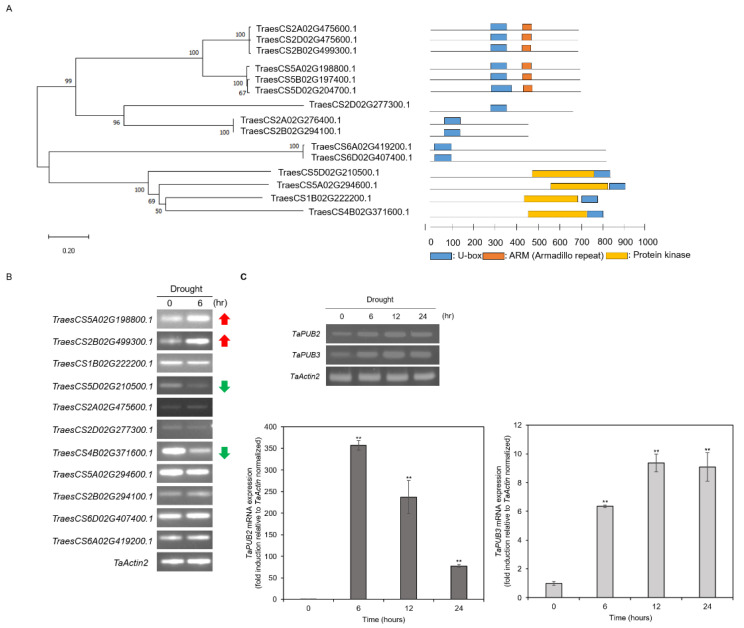
Identification and expression patterns of *TaPUB* genes. (**A**) Phylogenetic tree showing deduced protein sequences of 15 *TaPUB* genes. (on the right) Schematic diagram of full-length *TaPUB* cDNA. (**B**) RT-PCR of 11 *TaPUB* genes in wheat leaf during drought stress treatment (polyethylene glycol (PEG) (20 %) with different times (0 and 6 h)). Red and green arrows represent the up and downregulation of genes. (**C**) RT-PCR and qRT-PCR analyses of *TaPUB2* and *TaPUB3* in wheat leaf tissues treated with drought stress (PEG (20%) in a time course experiment (0, 6, 12, and 24 h)). The relative expression levels of genes are presented as the mean ± SD of three experimental replicates. *TaActin2* was used as the control. Asterisks represent statistically significant differences based on a two-tailed Student’s t-test when compared to controls (0 h); ** *p* < 0.01.

In a previous report, 213 PUB genes were identified and classified in wheat, according to their functional domain. Among them, 118 genes showed drought- and heat-stress-specific expression patterns [18]. Based on these results, we selected 15 genes that were highly expressed under drought stress conditions to identify the involvement of TaPUBs in the metabolism of drought stress responses (Figure 1A). Phylogenetic analysis revealed five groups, of which two groups consisted of three genes from each subgenome—A, B, and D. Furthermore, RT-PCR was used to examine the expression patterns of 11 U-box genes that were expressed under drought conditions, and the results indicated that the expression of two genes (*TraesCS5A02G198800.1* and *TraesCS2B02G499300.1*) was upregulated, while the expression of two genes (*TraesCS5D02G210500.1* and *TraesCS4B02G371600.1*) was downregulated after 6 h of drought treatment (Figure 1B). Therefore, *TraesCS5A02G198800.1* and *TraesCS2B02G499300.1*, named *TaPUB2* and *TaPUB3*, respectively, were selected for further studies. Furthermore, we tried to confirm the transcript levels of *TaPUB2* and *TaPUB3* in wheat leaf tissues treated with drought stress for different durations (0, 6, 12, and 24 h) (Figure 1C). Compared to *TaPUB2* and *TaPUB3* levels in the control (0 h), *TaPUB2* and *TaPUB3* were significantly induced in the leaves after 6 to 24 h of drought stress treatment.

### 2.2. TaPUB2 and TaPUB3 in Response to Different Abiotic Stresses

**Figure 2:**
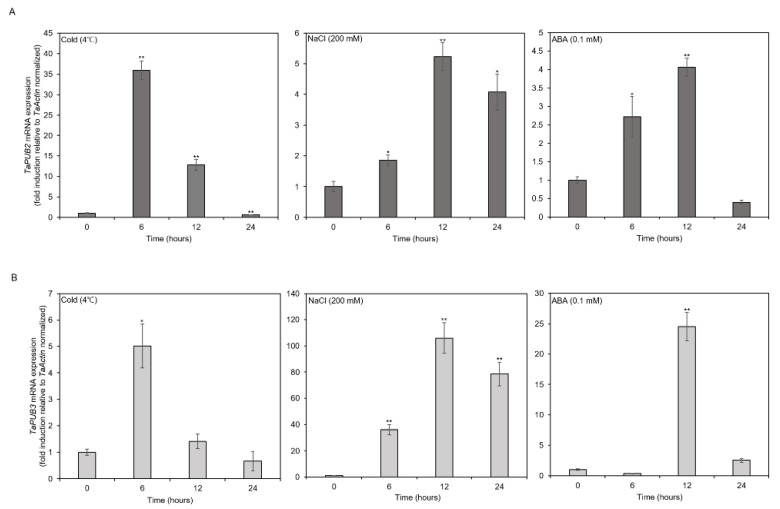
Gene expression patterns of *TaPUB2* and *TaPUB3* in response to different abiotic stresses (cold (4 °C), salt (200 mM NaCl), and abscisic acid (ABA) (0.1 mM)). (**A**,**B**) qRT-PCR analysis of *TaPUB2* and *TaPUB3* under different types of abiotic stresses in wheat leaf tissues. The TaPUB2 and TaPUB3 mRNA expression levels of the genes are presented as the mean ± SD of three biological replicates. *TaActin2* was used as the control. Asterisks represent statistically significant differences based on a two-tailed Student’s t-test when compared to controls (0 h); * *p* < 0.05, ** *p* < 0.01.

We analyzed the transcript levels of *TaPUB2* and *TaPUB3* in response to cold stress (4 °C), salt stress (200 mM NaCl), and ABA (0.1 mM) at 0, 6, 12, and 24 h to determine whether they were upregulated (Figure 2). The expression levels of *TaPUB2* and *TaPUB3* were highly increased under cold treatment at 6 h and decreased afterwards. *TaPUB2* and *TaPUB3* were highly expressed between 6 and 24 h under salt treatment. However, they were highly expressed only at 12 h after ABA treatment.

### 2.3. Subcellular Localization of TaPUB2 and TaPUB3 Proteins in Wheat Protoplast

**Figure 3:**
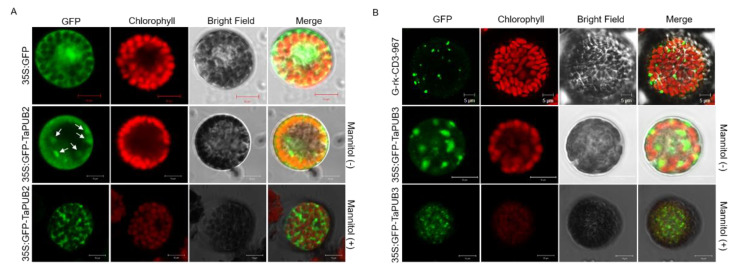
Subcellular localization of TaPUB2 and TaPUB3. Subcellular localization of (**A**) 35S:GFP-TaPUB2 and (**B**) 35S:GFP-TaPUB3 in wheat protoplasts. 35S:GFP and G-rk-CD3-967 were used as controls. A confocal laser scanning microscope was used to detect the fluorescence signal.

To examine the transient expression of *TaPUB2* and *TaPUB3*, the fluorescence signals of 35S:TaPUB2-GFP and 35S:TaPUB3-GFP were detected, respectively, in wheat protoplast cells using the polyethylene glycol (PEG) transfection system (Figure 3). The 35S:GFP-fused TaPUB2 was targeted to the cytosol and Golgi apparatus (Figure 3A). The 35S:GFP and Golgi apparatus marker (G-rk-CD3-967) were used to confirm the localization of TaPUB2 (Figure 3A,B) [27]. Similarly, the 35S:GFP-fused TaPUB3 was also targeted to the Golgi apparatus (Figure 3B).

### 2.4. In Vitro Ubiquitination Assay of TaPUB2 and TaPUB3

**Figure 4:**
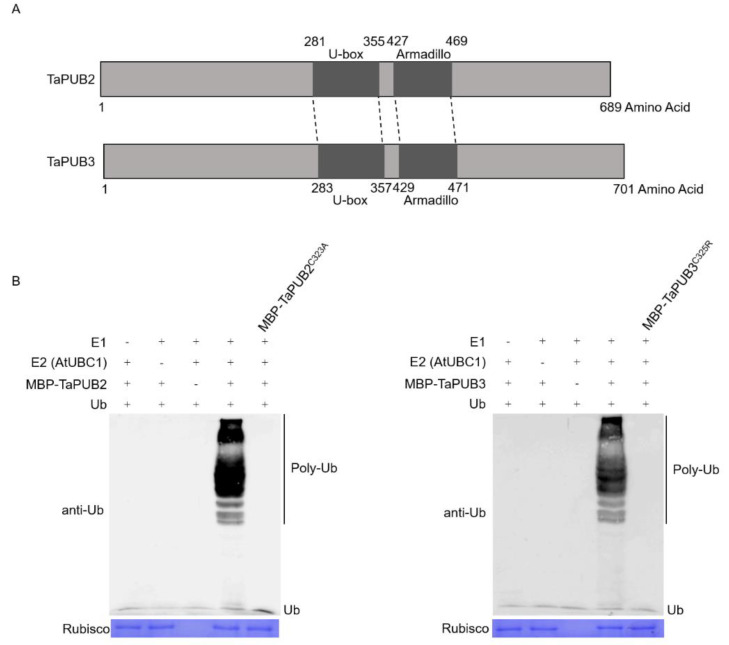
In vitro E3 ligase activity of TaPUB2 and TaPUB3. (**A**) Schematic diagram of full-length TaPUB2 and TaPUB3 proteins. The solid bar depicts the protein length. The U-box and Armadillo repeat domain are shown as dark gray bars. (**B**) The ubiquitination reaction contains E1 (human), E2 (AtUBC1), E3 (MBP-TaPUB2, MBP-TaPUB2^C323A^, MBP-TaPUB3, and MBP-TaPUB3^C325R^), and ubiquitin. Polyubiquitin chains are visible by immunoblotting with a ubiquitin antibody. (+) means presence, and (-) means absence.

*TaPUB2* (*TraesCS5A02G198800.1*) and *TaPUB3* (*TraesCS2B02G499300.1*) are homologous genes that encode putative E3 ligases with 61% amino acid sequence similarity in wheat (Figure 4A). Both proteins contain a U-box and an Armadillo (ARM) repeat domain in the central regions. Moreover, most U-box-containing proteins have been shown to possess E3 ligase activity. We performed an ubiquitination assay using MBP-tagged recombinant proteins (pMAL-c5x-TaPUB2 and pMAL-c5x-TaPUB3). The analysis showed that MBP-fused TaPUB2 and TaPUB3 generated poly ubiquitinated chains in the presence of E1, E2, and Ub (Figure 4B). Furthermore, we generated mutants—MBP-TaPUB2^C323A^ and MBP-TaPUB3^C325R^ to confirm the ubiquitination activity of the U-box domain, where cysteine-323 and cysteine-325 were replaced by alanine and arginine, respectively. However, no poly ubiquitinated chains were detected in the absence of E1, E2, E3, or mutated TaPUB2 and TaPUB3.

### 2.5. TaPUB2 and TaPUB3 Form Heterodimers

**Figure 5:**
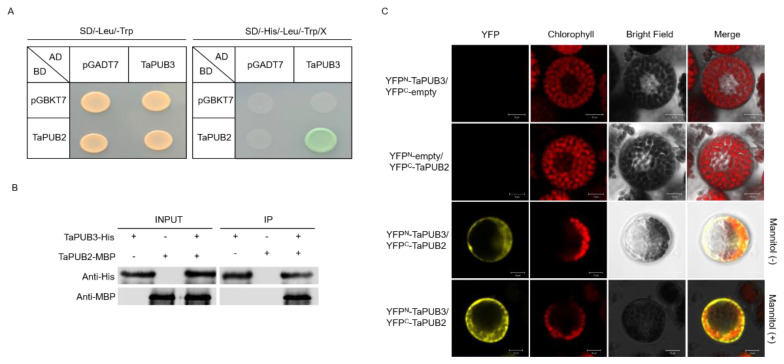
Heterodimeric complex formation of TaPUB2 and TaPUB3. (**A**) Yeast two-hybrid assay with TaPUB2 and TaPUB3. Full-length cDNA fragments encoding TaPUB2 and TaPUB3 were fused to sequences encoding GAL4 DNA-binding domain (BD) and GAL4 activation domain (AD) in pGBKT7 and pGADT7, respectively. Each construction was co-transformed into AH109 yeast strain in SD/-Leu/-Trp and SD/-Lue/-Trp/-His/-Ade/X-a-gal media. (**B**) In vitro pull-down assay of TaPUB3-His protein and MBP-TaPUB2 protein. (**C**) BiFC assay of TaPUB2 and TaPUB3 interactions using protoplast cells. TaPUB2 was fused into *C*-terminal YFP, and TaPUB3 was fused into *N*-terminal YFP. A confocal laser scanning microscope was used to detect the fluorescence signal.

The U-box/RING type E3 ligase often has a heterodimeric form, which has significant E3 ligase activity [28,29]. We performed yeast co-transformation to determine whether TaPUB2 and TaPUB3 form a complex. The full-length ORFs of TaPUB2 and TaPUB3 were cloned into the pGBKT7 (BD) and pGADT7 (AD) vectors, respectively, and co-transformed into AH109 yeast strain cells. Transformed yeast cells were placed onto D-aspartate oxidase DDO (SD/-Lue/-Trp) and QDO/X (SD/-Lue/-Trp/-His/-Ade/X-a-gal) growth media. The results showed that co-transformed TaPUB2 and TaPUB3 could interact with each other and form a heterodimer in quadruple dropout QDO/X media (Figure 5A). Next, we performed an in vitro pull-down assay using His-tagged TaPUB2 and MBP-tagged TaPUB3. The in vitro pull-down assay detected TaPUB3 protein, which was pulled down by TaPUB2 (Figure 5B). Furthermore, we examined the possible mechanisms by which TaPUB2 interacts with TaPUB3 using BiFC assays *in planta.* Both nYFP-TaPUB3 and cYFP-TaPUB2 were co-localized in the cytoplasm of the wheat protoplast cells (Figure 5C). These results implied that TaPUB2 and TaPUB3 bonded to each other to form a heterodimeric complex in yeast and plants.

### 2.6. Overexpression of TaPUB2 and TaPUB3 Confers Tolerance to Drought Stress in Arabidopsis Plants

**Figure 6:**
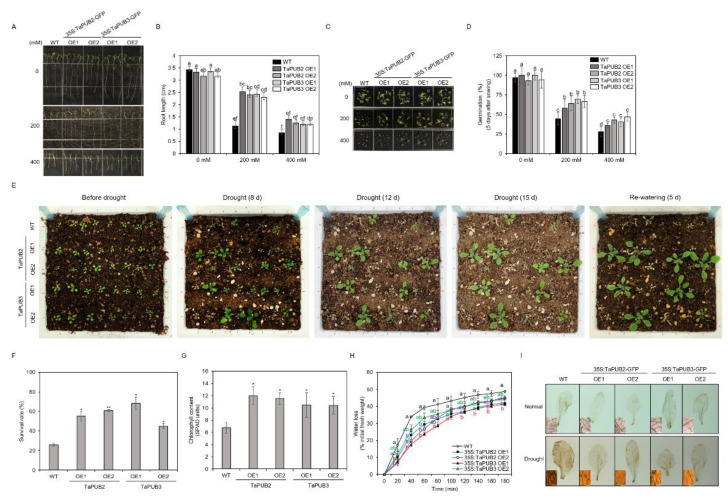
Phenotypes of WT and *35S:TaPUB2* and *35S:TaPUB3* overexpression plants in response to drought treatment. (**A**,**B**) Root length of the indicated genotypes. Four-day-old seedlings were transferred from 1/2 MS to 1/2 MS medium previously infused with different concentrations of mannitol (200 and 400 mM). Photographs were taken 4 days after the transfer. (**C**,**D**) Germination rate of *TaPUB2*- and *TaPUB3*-overexpressing plants in MS and mannitol (200, 400 mM)-containing MS media. (**E**) *TaPUB2*, *TaPUB3,* and WT grown under drought stress. After planting in soil, seedlings were not watered for 2 weeks and then re-watered for 1 week. (**F**) Survival rates (%) under drought conditions were determined as the number of visibly green plants after rehydration. (**G**) Total chlorophyll content was measured in WT and overexpressing plants after recovery. (**H**) Comparison of the rate of water loss from detached rosettes between WT and overexpressing plants. (**I**) H_2_O_2_ content in *Arabidopsis* under normal conditions and drought treatment, with 3,3-diaminobenzidine staining assay of rosette leaves of WT and *TaPUB2* and *TaPUB3*-overexpressing lines. Values are mean ± SD, n = 3. * Indicates significant difference between *TaPUB2* or *TaPUB3* plants and WT control based on the ANOVA (* 0.01 ≤ *p* < 0.05, ** *p* < 0.01), using the Duncan’s multiple range test (DMRT). Different letters indicate significant differences (*p*-value 0.05) among the genotypes under the same treatment.

To evaluate the molecular function of TaPUB2 and TaPUB3 under drought conditions, we developed *TaPUB2-* and *TaPUB3*-overexpressing *Arabidopsis* plants, respectively (Figure S1). Heterologous overexpression of *TaPUB2* and *TaPUB3* led to a higher percentage of seed germination rate than that of the wild type (WT) upon treatment with different concentrations of mannitol (200 and 400 mM) on a half-strength MS medium (Figure 6C,D). Similarly, we measured the root lengths in transgenic and WT plants that had been exposed to different concentrations of mannitol (200 and 400 mM) for 4 days after transferring 4-day-old seedlings onto 1/2 MS media. The root lengths of *TaPUB2* (OE1 and OE2) and *TaPUB3* (OE1 and OE2) transgenic plants significantly increased compared with that of the WT, while the root length under normal growth conditions did not differ clearly between the two groups (Figure 6A,B).

The performance of soil grown *TaPUB2* and *TaPUB3* transgenic seedlings was examined under water deprivation conditions in the soil. Under normal conditions, plants did not exhibit any morphological differences between the WT and OE lines. After 15 days of normal growth conditions, the plants were deprived of water for 14 days and then watered normally (Figure 6E). *TaPUB2* and *TaPUB3*-overexpressing plants showed a significantly higher survival rates (by 2.3-fold) compared with that of the WT plants (Figure 6F). Furthermore, under drought stress conditions, the chlorophyll content of the transgenic plants was 1.5-fold higher than that of the WT plants (Figure 6G). In contrast, *TaPUB2* and *TaPUB3*-overexpressing plants were found to have a lower water loss rate than that of the WT plants (Figure 6H).

Additionally, the accumulation of ROS in *TaPUB2* and *TaPUB3* transgenic lines and WT plants was analyzed using 3,3-diaminobenzidine (DAB) staining and nitroblue tetrazolium (NBT) analysis. Under drought stress conditions, tthe WT plants accumulated more ROS than the OE lines (Figure 6I and Figure S2). These results strongly suggested that *TaPUB2* and *TaPUB3* function in the same signaling pathway in their plant responses to drought stress.

### 2.7. Transcriptional Analysis of Drought Stress Responsive Genes in TaPUB2 and TaPUB3-Overexpressing Plants

**Figure 7:**
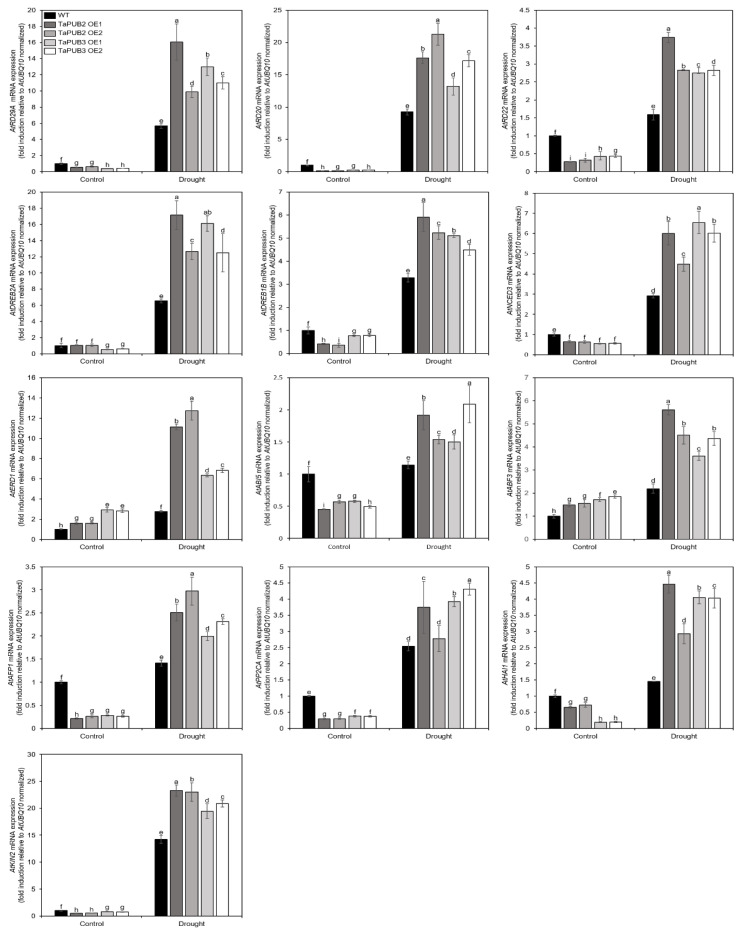
Real-time qRT-PCR analysis of drought-stress related maker genes. For dehydration treatment, the lid of the dish was opened under light for 5 h at room temperature. The induction patterns of various ABA- and/or drought-responsive genes (*RD29A, RD20, RD22, DREB2A, DREB1B, NCED3, ERD1, ABI5, ABF3, AFP1, PP2CA, HAI1*, and *KIN2*) were analyzed using qRT-PCR. Data represent the fold induction of each gene under dehydration (5 h) relative to the gene level of the control treatment (0 h). The mean values from three independent technical replicates were normalized to the levels of the internal control, *UBC10* mRNA. Different letters indicate significant difference at (*p* < 0.05) between the WT and *35S:TaPUB2* and *35S:TaPUB3 Arabidopsis* transgenic plants.

To verify the mRNA level changes of several drought stress-induced genes in *TaPUB2* and *TaPUB3* transgenic plants, we performed qRT-PCR under drought (air drying) conditions. The overexpression of *TaPUB2* and *TaPUB3* in *Arabidopsis* plants showed a tolerant phenotype under drought conditions (Figure 6). Therefore, to evaluate the transcript expression of drought-induced genes, we examined 13 drought-responsive genes, including *AtRD29A* (AT5G52310), *AtRD22* (AT5G25610), *AtRD20* (AT2G33380), *AtDREB1B* (AT4G25490), *AtDREB2A* (AT5G05410), *AtNCED3* (AT3G14440), *AtERD1* (AT5G51070), *AtABF3* (AT4G34000), *AtAFP1* (AT1G69260), *AtPP2CA* (AT3G11410), *AtHAI1* (AT5G59220), *AtABI5* (AT2G36270), and *AtKIN2* (AT2G02800) [30,31,32,33,34]. Statistical analysis of these genes’ mRNA expression levels revealed a significantly increased transcription level when compared to their WT counterparts during drought conditions (5 h), while some of the genes in fold induction level could be detected under control conditions (Figure 7). Furthermore, *G*, *T*, and *G* × *T* had a significant impact on the expression of these genes (Table S2). These results revealed that *TaPUB2* and *TaPUB3* positively regulated the transcript levels of these genes under drought conditions.

## 3. Discussion

Although the role of PUB E3 ligases has been reported in many abiotic and biotic stress conditions in various plant species, their molecular and metabolic functions in wheat remain unclear, particularly under abiotic stress conditions [19,35,36], since their biological and molecular study has only been in the initial stages in wheat. In this study, we identified functional analysis of two PUB E3 ligases, TaPUB2, and TaPUB3, in wheat (Figure 1A and Figure 4A). The transcriptional profiles of *TaPUB2* and *TaPUB3* were confirmed by the results of their response to abiotic stresses in wheat; the expression of both *TaPUB2* and *TaPUB3* was highly upregulated under abiotic stresses—drought, cold, salt, and ABA (Figure 1 and Figure 2). Furthermore, the sequence similarity index (61%) and domain structure analysis revealed the presence of a conserved domain (ARM and U-box) and other residues, indicating that TaPUB2 and TaPUB3 were highly conserved (Figure 4A). Subcellular localization analysis revealed that TaPUB2 and TaPUB3 were localized in the Golgi apparatus (Figure 3A), indicating that they may have been involved in various intercellular mechanisms or functions in wheat.

Several studies have reported that wheat U-box proteins have multiple functions in response to abiotic stresses. For example, the U-box protein TaPUB1 improved salt and drought stress tolerance in *Nicotiana benthamiana* [13]. *TaPUB15* overexpression resulted in enhanced root growth and development and an increased resistance to salt stress in rice [37]. E3 ubiquitin ligase functions in response to abiotic stress and is dependent on the ABA pathway [38]. Similarly, TaPUB1 and TaPUB15 E3 ligases were shown to be highly expressed in transgenic plants in response to ABA signaling and enhance tolerance to salt stress [13,37]. In our study, the transcription of *TaPUB2* and *TaPUB3* was significantly induced by drought stress and highly upregulated by ABA, cold, and salt stresses (Figure 1 and Figure 2), suggesting that TaPUB2 and TaPUB3 may be involved in drought, cold, and salt stress responses in an ABA-dependent manner. The bZIP transcription factor (TFs) are well known drought stress response pathways. bZIPs members are related in post-translational modification, such as phosphorylations/de-phosphorylation events [39]. bZIP TFs, including nine subfamily member ABREs/ABFs, have existed in *Arabidopsis.* Among them, ABF1, AREB1/ABF2, AREB2/ABF3, and ABF4 have been phosphorylated by SnRK2s (Snf1-related protein kinase) and regulate the expression of ABA-dependent drought signaling pathways [40]. In this study two ABA-dependent drought induced genes, such as *AtABI5* and *AtABF3*, were up-regulated in response to the *TaPUB2* and *TaPUB3*-overexpressing in plants growing drought condition (Figure 7). Moreover, when there was a water deficit condition, ABA caused stomatal closure, reducing water loss through transpiration [41]. These results were consistent with reducing the water loss rate in *TaPUB2* and *TaPUB3-overexpressing plants* (Figure 6H). However, further work is needed to understand the ABA-dependent response and other abiotic stress mechanisms in transgenic plants.

A previous study [26] demonstrated that heterologous overexpression of *TaPUB1* positively regulates post-germination plant growth under drought stress (PEG 6000). To study the physiological function of *TaPUB2* and *TaPUB3* in plant growth as well as drought tolerance, we generated transgenic Arabidopsis plants using the floral dipping method (Figure S1). Water deficit significantly delayed the onset of seed germination rate. Some of E3 ligase, which contains the U-box domain, have important roles in seed germination under the drought stress [42]. Similarly, under drought stress conditions, the seed germination rate of TaPUB2 and TaPUB3 overexpression plants was much greater than that of WT plants (Figure 6C,D). Moreover, the root development of the *TaPUB2* and *TaPUB3* transgenic lines have also longer than WT under osmotic stress conditions (Figure 6A,B). Water deficiency has a considerable impact not only during the seedling stage but also during the vegetative stages [26]. Under drought stress, *TaPUB2* and *TaPUB3* transgenic plants showed improved survival rates and robust phenotypes, such as green leaves and high chlorophyll content (Figure 6). These findings indicate that U-box type E3 ligases were involved in the regulation of plant adaptation mechanisms during harsh environments, such as drought.

When the amount of available soil water is severely limited, the first option for plants induced accumulation of ROS and close their own stomata [8]. Drought stress causes oxidative stress by increasing ROS, such as ^1^O_2_ (singlet oxygen), O_2_^−^ (superoxide radical), HO• (hydroxyl radical), and H_2_O_2_ (hydrogen peroxide), all of which are highly reactive and contribute to drought stress [43]. Excess ROS are scavenged or detoxified by an efficient antioxidative system that includes both nonenzymic and enzymic antioxidants, such as superoxide dismutase (SOD), catalase (CAT), and ascorbate peroxidase (APX) [44]. Previously, [45] it was demonstrated that drought stress resistance in *VyP5CR* transgenic *Arabidopsis* revealed less efficient scavenging of H_2_O_2_ as compared WT lines. In the present study, we observed that *TaPUB2* and *TaPUB3* transgenic line have a more effective antioxidant defense system and less accumulation of ROS (Figure 6I and Figure S2). These findings revealed that TaPUB2 and TaPUB3 were involved in mechanisms that protect cells during drought stress.

The predicted U-box protein has a domain similar to that of the RING-finger protein, despite the loss of hallmark and histidine residue [46,47], and is found in combination with other domains, such as ARM (armadillo) repeats, Ser/Thr kinase domain, WD40 repeats, or tetratricopeptide (TPR) domain, or peptidyl-prolyl isomerase [48]. E3 ligase, which contains a U-box domain, performs ubiquitination upon forming a complex with the activating enzyme E1 and conjugating enzyme E2 [49]. We incorporated a single amino acid mutation in *TaPUB2* and *TaPUB3* by substituting cysteine at the 323rd position with alanine and cysteine at the 325th position with arginine, respectively, in the U-box domain. A previous report demonstrated that PUB E3 ligase activity depends on cysteine residues [15]. Thus, the mutations in *TaPUB2* and *TaPUB3* result in catalytically inactive forms in the presence of E1, E2, and ubiquitin (Figure 4B). These findings showed that TaPUB2 and TaPUB3 have E3 ligase activities, and they require the U-box domain to perform ubiquitination.

Recent studies have shown that several E3 ligases play combinatory roles, suggesting that they form heterodimers [39,40]. Furthermore, [50] identified homologous OsPUB2 and OsPUB3 proteins degrade rapidly upon forming a heterodimer. Therefore, we tested whether TaPUB2 interacts with TaPUB3 using the yeast-two hybrid assay (Figure 5A). Moreover, we confirmed the interaction between TaPUB2 and TaPUB3 using BiFC assays (Figure 5B). Interestingly, the TaPUB2/TaPUB3 protein complex occurred in the cytoplasm, whereas TaPUB2 was transiently expressed, and TaPUB3 was localized in the Golgi apparatus (Figure 3). The spatial differences in the expression of complex proteins could also be observed in other studies. For example, the cytoplasm and nucleus-localized Histone H2B mono-ubiquitination enzyme (TaHUB2) physically interacts with the nuclear proteins, such as TaELF7, in wheat [51]. OsSADR1 interacts with and regulates the chloroplast-localized protein OsPIPIN in the cytoplasm [8]. Therefore, TaPUB3 might be translocated from the Golgi apparatus to the cytoplasm, thus interacting with TaPUB2 to form a heterodimeric complex.

## 4. Materials and Methods

### 4.1. Plant Material and Growth Conditions

The seeds of a wheat variety [*Triticum aestivum* L. ‘Keumkang’ (Korea RDA Accession No. IT213100)] were germinated and grown in a growth chamber (25 °C with 60% relative humidity and photoperiod of 16 h light intensity) on mesh supported in plastic containers with one-half concentration of MS (Murashige and Skoog) liquid media. The 14-day-old plants were treated with abiotic stresses, including drought (20% PEG 6000), salt (200 mM), cold (4 °C), and ABA (0.1 mM). Leaf tissues were collected at 0, 6, 12, and 24 h following stress treatment. Samples were immediately frozen in liquid nitrogen for further experiment.

### 4.2. Gene Cloning and Phylogeny Analysis

PUB protein sequences were obtained from a previous study by [18]. The ORFs of *TaPUB2* (*TraesCS5A02G198800.1*) and *TaPUB3* (*TraesCS2B02G499300.1*) were amplified using primer sets designed based on the wheat reference sequence Ensembl Plants (https://plants.ensembl.org/index.html (accessed on 16 December 2021)). i-pfu DNA polymerase (iNtRON Biotechnology, Cat: 25181, Seongnam, Gyeonggi, Korea) and primers were used for PCR amplification (Table S1). TaPUB2 and TaPUB3 were cloned and sequenced into the pCR8/GW/TOPO vector (Invitrogen, Cat: K250020, Carlsbad, CA, USA). MEGA-X software was used to generate a phylogenetic analysis [52].

### 4.3. Gene Expression Studies

RNA extraction, cDNA conversion, primer design, and qRT-PCR was used to analyze the transcript expression patterns of the putative genes and the results were estimated using the delta-delta CT method [51,53]. qRT-PCR experiments were performed from three independent biological replicates.

### 4.4. Subcellular Localization

To examine the subcellular localization of TaPUB2 and TaPUB3, two genes were cloned into the GFP vector (pMDC43) and BiFC vector (pGTQL1211 and pGTQL1221) using LR reaction (Invitrogen, Cat: 11791020, Carlsbad, CA, USA), respectively. The resulting constructs were used for transient assays using wheat protoplasts transfected with polyethylene glycol [54].

### 4.5. Ubiquitination Assay

To perform the E3 ligase activity of TaPUB2 and TaPUB3, each gene was cloned into the MBP-tagged vector (pMAL-c5x). An in vitro ubiquitination experiment was carried out using a previously described protocol [10,55].

### 4.6. Yeast Two-Hybrid Assay

The Matchmaker GAL4 bases two-hybrid system was used for the yeast two-hybrid assays (Clontech, Cat: 630489, Palo Alto, CA, USA). The full-length sequences of *TaPUB2* and *TaPUB3* were recombined into the pGDAT7 (including activation domain (AD)) and pGBKT7 (including binding domain (BD)), respectively. *TaPUB2*, *TaPUB3,* and empty vector were co-transformed and grown in DDO medium after being transformed into the AH109 yeast strain. Each clone was cultured in DDO broth for 5 days before being spotted on a QDO medium to test for protein-protein interactions.

### 4.7. Pull-Down Assay

To determine the PUB2 physical interaction with TaPUB3, MBP-tagged TaPUB2 and His-tagged TaPUB3 proteins were expressed in *Escherichia coli* (strain BL21). The pull-down assay was used for His Protein Interaction Pull-Down Kit (Invitrogen, Cat: 21277, Carlsbad, CA, USA), as previously described by [54].

### 4.8. Generation of Transgenic Arabidopsis Plants

For overexpression of *TaPUB2* and *TaPUB3* in *Arabidopsis*, the ORF fragments that had been amplified were subcloned into pMDC43 vectors. The resulting plasmids were introduced into pMDC43 binary vectors to obtain the *35S:TaPUB2-GFP* and *35S:TaPUB3-GFP* constructs that were used for *Agrobacterium* (GV3101)-mediated transformation in *Arabidopsis* (ecotype Columbia-0) using the floral dipping method [56]. All experiments were carried out on homozygous T3 generation plants. For each assay, at least two independent transgenic lines were used.

### 4.9. Dehydration and Drought Tolerance

Overexpression lines and WT seeds were germinated and grown for 15 days at 25 °C in a growth chamber (25 °C with 60% relative humidity and photoperiod of 16 h light intensity). Some seedlings were dehydrated, while others were subjected to drought treatment by stopping irrigation for 14 days. The rosette leaves of 14-day-old seedlings of each transgenic line were placed on filter papers and allowed to dry for up to 5 h for dehydration analysis. To determine the rate of water loss, the fresh weight of each sample was measured every 20 min. To detect H_2_O_2_, the leaves were incubated for 4 h in a 1 mg/mL DAB solution (Sigma-Aldrich, Cat: D8001, St Louis, MO, USA). Following the incubation period, the leaf samples were immersed in a bleaching solution (ethanol: acetic acid: glycerol, 3:1:1) and boiled for 15 min at 95 °C [50].

### 4.10. Statistical Analysis

All experimental data were the means of at least three separate replicates, and statistical analysis was carried out with Microsoft Excel 2016. (Microsoft, Seattle, WA, USA). For comparisons between two analyzed populations and among three or more populations, the Student’s t-test and Duncan’s multiple range test from one-way and two-way analysis of variance (ANOVA) were used to determine if the differences were statistically significant at *p*-values < 0.05.

## 5. Conclusions

In the present study, the molecular function of TaPUB2 and TaPUB3, including their expression patterns, subcellular localization, and E3 ligase activity was elucidated. Two proteins directly interact with one another and form heterodimeric complexes (TaPUB2/TaPUB3). Furthermore, TaPUB2 and TaPUB3-overexpressing *Arabidopsis* displayed a resistance phenotype under the drought condition (media and soil) and a lesser rate of ROS accumulation, respectively. These findings suggest that the two putative U-box E3 ligases, TaPUB2 and TaPUB3, are involved in the response to abiotic stresses, especially drought. However, studying double mutants or transgenic wheat lines would be helpful to further understand the genetic mechanisms involved in drought tolerance.

## Data Availability

This research did not report any data.

## Supplementary Materials

The following are available online at https://www.mdpi.com/article/10.3390/ijms222413658/s1.
