# Supplementary material for: Molecular Characterization of U-box E3 Ubiquitin Ligases (TaPUB2 and TaPUB3) Involved in the Positive Regulation of Drought Stress Response in Arabidopsis
Source: Int J Mol Sci. 2021 Dec 20;22(24):13658. doi: 10.3390/ijms222413658 (PMC8704797; doi:10.3390/ijms222413658)
Supplement: Supplementary file 1 [file ijms-22-13658-s001.zip › ijms-1490252-supplementary.pdf]

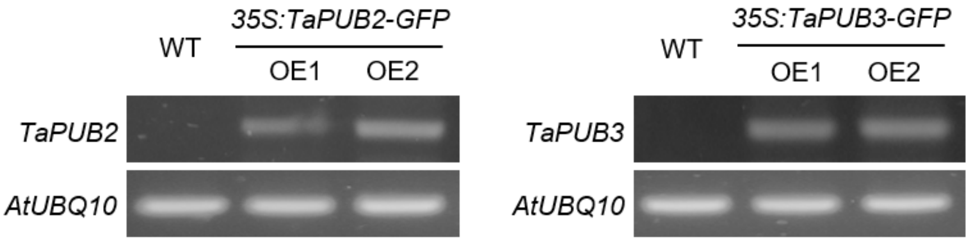

Figure S1. Semiquantitative RT-PCR of *TaPUB2*- and *TaPUB3*-overexpressing *Arabidopsis* and control plants

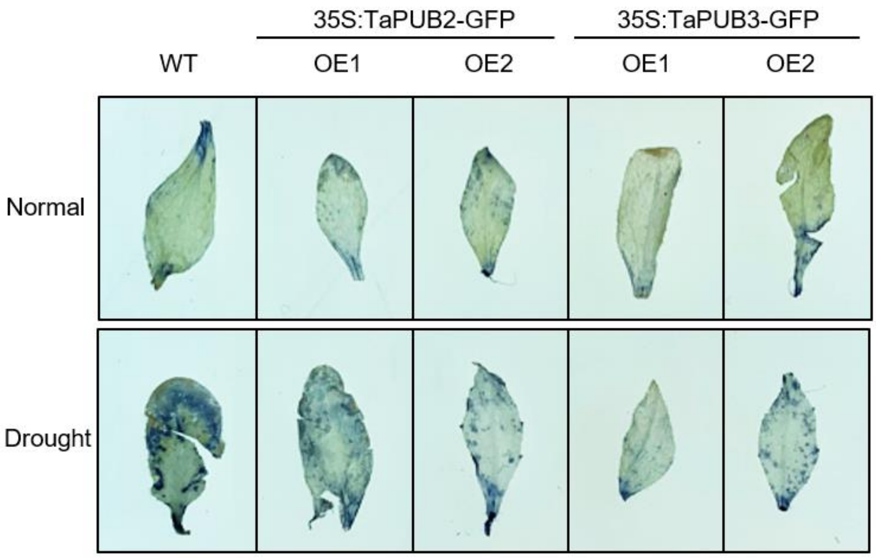

Figure S2. H_2O_2 content in *Arabidopsis* under normal conditions and drought treatment. nitroblue tetrazolium staining assay of rosette leaves of WT and *TaPUB2* and *TaPUB3*-overexpressing lines

Table S1. The PCR primers used in this study.

Gene	Accession	Purpose (cloning)	Forward (5'à3')	Reverse (5'à3')
TaPUB2	TraesCS2B02G499300.1	PCR8/GW/Topo	ATGCCGCCGCACGACCGTAAG	TCGAATGACACGGTCACCCGC
		Y2H (pGADT7, pGBKT7)	GAGACATATGATGCCGCCGCA	GAGAGAATTCTACTCGAATGACACGGTCAC
		ubiquitination assay (pMAL-c5x)	GAGACATATGATGCCGCCGCA	GAGAGAATTCTACTCGAATGACACGGTCAC
		Mutation	AGGGCGGGTGCCGGACGTGCCCTGTTACCG	TTCTCGCCGGTAACAGGGCACGTCCGGCAC
		Real-time PCR	TTTTCACAAGCCTCGTCGACTT	CATAAGCGCAGTCATCTTGCC
TaPUB3	TraesCS5A02G198800.1	PCR8/GW/Topo	ATGCCGGCCCCGCCGCCGCCA	TAGCACGAGCCGAACGAAA
		Y2H (pGADT7, pGBKT7)	GAGACATATGATGCCGGCCCCGC	GAGAGAATTCTAGCACGAGCCGAACGAAACGGTC
		ubiquitination assay (pMAL-c5x)	GAGACATATGATGCCGGCCCCGCCG	GAGAGAATTCTAGCACGAGCCGAACGAAACGGTCGCC
		Mutation	AAGGCCGGGTGCCGCACGCGCCCAGTCACCGGC	CTTCTCGCCGGTGACTGGGCGCGTGCGGCACCC
		Real-time PCR	TCCTCCTCAACAATCTGATGGC	TCATGAGTTCCAGTGTGATCGG
TaActin	Ta54227	Real-time PCR	CAAATACGCCATCAGGGAGAACATC	CGCTGCCGAAACCACGAGAC
TaPUBs	TraesCS5D02G210500.1	Real-time PCR	TACTTCCTCTGCCCGATACTCA	GGGAAGCTTGAGGTTGGTCATC
	TraesCS2A02G475600.1		GACACCGTTGATGCCAAGAAAG	TTGATCCACCGTTTGATGGACG
	TraesCS2D02G277300.1		TGTGCCCTTTCTCCAAGAAGTT	ACTGGACACATGCGTTTGTTTT
	TraesCS4B02G371600.1		CATCCAAGGACGCTGATAGGAA	ACCGTTCCATTAGACCCAGTTC
	TraesCS5A02G294600.1		AGGATTATGCAAGGTTCCAGCA	GCAGGACTGTTCTTCTCGATCA
	TraesCS2B02G294100.1		ATCACAAATGACCTTTGCACGG	GATTGCTTCTGGGTCATCTCCA
	TraesCS6D02G407400.1		GCATTGGCTAACGATGTGAAGG	CTCGTTGGATGACATCTCCCTC
	TraesCS6A02G419200.1		TCTCCTCACCAAACTGCTTCAA	GATGCTCACCAGAAGAGTTCCA
	TraesCS1B02G222200.1		ATCTGCCCAATTTCTCAGGAGG	ATTCTGTATGGCGGAACGAAGA
AtUBC1	At1G14400	ubiquitination assay (pET28a)	GAGAGGATCCATGTCGACGCCAGCAAGGAAG	GAGAAAGCTTGGTCAGCAGTCCAGCTTTGCTCA
AtNCED3	At3G14440	Real-time PCR	CCAGATTGCTTCTGCTTCCAT	GACCCTATCACGACGACTTCATC
AtRD22	At5G25610		GAGCTAAAGCAGTTGCGGTATG	TCCCTGGCTTCACCTTTAAGAC
AtRD20	At2G33380		TTTAGCAAATACGCGCTAACGG	GATTGCCATTCGATTTCCCTCG
AtRD29A	At5G52310		CCTGAAGTGATCGATGCACCAG	TGGTGTAATCGGAAGACACGAC
AtERD1	At5G51070		CAGATGATGGAGATCTTGAA	TGCGATCGATGTTTTGT
AtDREB1B	At4G25490		CCAGTTTCTTGAAACAGAGTAC	GGTGACGAGTCTCACGAAACT
AtDREB2A	At5G05410		GAAGAAGAAAAGATACGAGCAAG	CGATGTATCAATTTGTGTTCTG
AtABF3	AT4G34000		CTTTGTTGATGGTGTGAGTGAG	GTGTTTCCACTATTACCATTGC
AtAFP1	AT1G69260		ATGGAGCCACAAGGGAAAGG	GAGGACCACCGGCATGTTTA
AtHAI1	AT5G59220		CCATGGCTGTTCCCATGTA	AAGCTACGCGCCATTGAC
AtPP2CA	AT3G11410		GGCGAGTGATGGACTATGGG	TTTGTTAAGAGCAACGCCGC
AtABI5	AT2G36270		AGATGACACTTGAGGATTTCTTGGT	TGGTTCGGGTTTGGATTAGG
AtKIN2	AT2G02800		GCAACAGGCGGGAAAGAGTAT	CCGGTCTTGTCCTTCACGAA
AtUBQ10	AT4G05320		CAAATACGCCATCAGGGAGAACATC	CGCTGCCGAAACCACGAGAC

Table S2. Analysis of variance (ANOVA) of the transcript responses of WT, TaPUB2 (OE1, OE2), and TaPUB3 (OE1, OE2) under non-treatment and drought treatment.

Parameters	Source of variations			
	Genotype (*G*) (*df*=9)	Treatment (*T*) (*df*=1)	$G \times T$ (*df*=9)	F
RD29A	**	**	**	363.639
RD20	**	**	**	706.098
RD22	**	**	**	32.632
DREB2A	**	**	**	118.064
DREB1B	**	**	**	35.708
NCED3	**	**	**	196.339
ERD1	**	**	*	2.695
ABI5	**	**	**	105.033
ABF3	**	**	**	69.073
AFP1	**	**	**	240.376
PP2CA	**	**	**	119.702
HAI1	**	**	**	300.284
KIN2	**	**	**	628.425
